# Cluster randomized, controlled trial on patient safety improvement in general practice: a study protocol

**DOI:** 10.1186/1471-2296-14-127

**Published:** 2013-08-29

**Authors:** Natasha J Verbakel, Maaike Langelaan, Theo JM Verheij, Cordula Wagner, Dorien LM Zwart

**Affiliations:** 1 Department of General Practice, Julius Center for Health Sciences and Primary Care, University Medical Center Utrecht, Utrecht, The Netherlands; 2 Nivel, Netherlands Institute for Health Services Research, Utrecht, The Netherlands; 3 EMGO+ Institute, Department of Public and Occupational Health, VU University Medical Center, Amsterdam, The Netherlands

**Keywords:** Patient safety, Safety culture, Questionnaire, Intervention, Trial

## Abstract

**Background:**

An open, constructive safety culture is key in healthcare since it is seen as a main condition for patient safety. Studies have examined culture improvement strategies in hospitals. In primary care, however, not much is known about effective strategies to improve the safety culture yet. The purpose of this study is to examine the effect of two patient safety culture interventions: a patient safety culture questionnaire solely, the SCOPE, or the SCOPE questionnaire combined with a patient safety workshop. The purpose of this paper is to describe the rationale and design of this trial.

**Methods/design:**

The SCOPE Intervention Study is a cluster randomized, three-armed controlled trial, that will be conducted in 30 general practices in the Netherlands. Ten practices in the first intervention arm will complete the SCOPE questionnaire and are expected to draw and implement their own improvement initiatives based on a computerised feedback report. In the second intervention arm, staff of the ten practices also will be asked to complete the SCOPE questionnaire and in addition will be given a complementary workshop. This workshop is theoretical and interactive, educating staff and facilitating discussion, leading to a practice specific action plan for patient safety improvement. The results of the SCOPE questionnaire are incorporated in the workshop. The ten practices in the control arm continue care as usual. Baseline and follow-up measurements will be conducted with an implementation period of one year. The primary outcome will include the number of incidents reported and secondary several quality and safety indicators and the patient safety culture. Moreover, interviews will be conducted at follow-up to evaluate the implementation process of the intervention.

**Discussion:**

Results of this study will give insight in the effect of administering a culture questionnaire or the questionnaire with a complementary workshop. This knowledge will aid implementation of patient safety tools and future research. Attention has been given to the strengths and limitations of the study.

**Trial registration:**

Netherlands Trial Register: NTR3277.

## Background

A main condition for patient safety is an open constructive safety culture. Patient safety culture is described as the values, attitudes, norms, beliefs, practices, policies, and behaviours regarding safety issues in daily practice [1]. One of the main recommendations in the Institute of Medicine report ‘to Err is Human’ was to support a safety culture. The National Patient Safety Agency in the UK also recognizes the importance of an open culture. In their developed "Seven steps to patient safety for primary care" the first step is to "build a safety culture" [2]. In a report about safety in healthcare in the Netherlands, the former director of Shell, called an environment where acknowledging mistakes is taboo, one of the main causes of safety-risks [3]. Non-medical industries have been working on safety for much longer and showed that an open culture on error ameliorates business performance [4,5]. Reports suggest a similar role of safety culture in healthcare [6,7].

In hospital care, team training and communication, executive walkrounds and Comprehensive Unit-Based Safety Program (CUSP) are well received interventions to improve patient safety culture that have been studied. Although positive effects were reported, the level of evidence moderates firm conclusions on the effectiveness of patient safety culture in healthcare [8,9]. Despite the fact that a large part of healthcare is delivered in primary care where practice organisations are becoming larger scaled and more complex, leading to increasing importance of patient safety issues, the effectiveness of such improvement strategies in primary care is underexposed.

Often, the first step to initiate patient safety culture improvements is to measure the current state of affairs. We have developed and validated a patient safety culture questionnaire for general practice: the SCOPE [10]. During this former study we observed that this culture questionnaire raised awareness and stimulated some professionals to change their practice. The conducting of a survey can be perceived as a measurement tool and also as a vehicle for communication. It is stated that the actual administration of a survey operates as an intervention. The survey affects people’s perceptions and sends messages to employees about the importance of the topic it addresses [11]. Also, feedback of patient safety culture surveys, combined with benchmark data are found highly informative [12]. Others observed a possible intervention effect of conducting a culture questionnaire [9,13,14].

However, it is not clear what the magnitude and the sustainability of the application of a single questionnaire is. It appears that professionals find it difficult to shape actual improvement in practice [15]. We expect that the effect of a single questionnaire could only be temporarily, and subsequently, that the raised awareness will fade away and thus will not lead to actual safety culture improvements. Sexton et. al developed a tool to discuss results of a culture questionnaire as they state that without such a tool it would be unlikely that spontaneous discussions lead to meaningful improvements in cultures, given the relative novelty of safety culture and its complexity [16]. This corresponds to the reasoning that “the process of reporting results is perhaps most important in determining a survey’s effectiveness as a cultural change tool” [11]. Hence, a more practical and comprehensive intervention seems needed for obtaining profound and lasting results. Following this, we develop a complementary workshop to the SCOPE questionnaire, based on the Manchester Patient Safety Framework (MaPSaF) [17]. The MaPSaF is developed by the NHS specifically for primary care. The tool aims at helping primary care practices to assess the current level of maturity (pathological, reactive, bureaucratic, proactive, generative) of their approach to patient safety. The tool’s output serves as a basis for discussions on how to improve the practices’ patient safety. Our approach resembles the CUSP, an eight step programme to improve safety culture, used in hospitals [18]. The first step is to measure the culture, followed by the science of safety, identification of safety concerns by staff, adopting of a unit by senior executives, implementation of improvements, analysing and documentation of efforts, sharing of results and last, reassessment of culture.

### Objectives

The first objective of this study is to examine the effect of two interventions on patient safety behaviour and patient safety culture in general practice: the SCOPE questionnaire solely, and the SCOPE questionnaire combined with a safety culture workshop. We conduct a three armed trial instead of a two-armed trial. The purpose of the ‘questionnaire only’ arm is twofold. Firstly, to assess whether administering a culture questionnaire with only a feedback report has an effect on patient safety behaviour and culture, compared to the control arm. Secondly, to be able to adjust for the possible intervention effect of the questionnaire in the workshop arm.

Our second objective is to evaluate the implementation process of both interventions. Designing, implementing and evaluating a patient safety culture intervention is complex. The direction of results will largely depend on the context [19-22]. Therefore, evenly important as the possible effect of the interventions is the process evaluation.

## Methods/design

### Design and setting

The SCOPE Intervention Study is a cluster randomized, three-armed controlled trial complemented with a qualitative study. The study will be conducted in thirty general practices in the Netherlands.

### Practices selection and randomization

**Figure 1:**
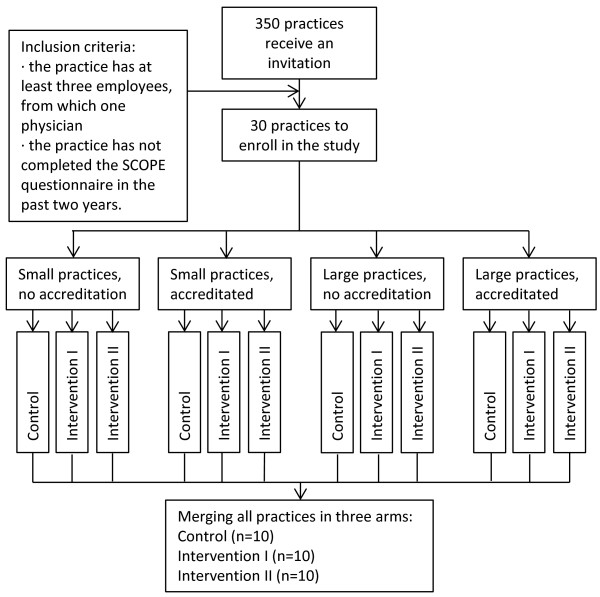
Flowchart randomisation.

All general practices (n = 350) in Utrecht area receive an invitation to participate in the study. Practices that consist of at least three employees of whom at least one GP can participate in the study. In addition, the SCOPE questionnaire should not have been completed in the past two years. Stratified randomization will be used to allocate the practices in the three trial arms (see Figure 1). Stratification is based on practice size and whether a practice is accredited on the Dutch GP Practice Accreditation system [23], as we expect these parameters to possibly confound the effects on patient safety culture. The randomisation will be performed by the Data Management Unit of the Julius Center, independent of the research team. Because of the nature of the intervention blinding is not feasible.

### Procedure

**Figure 2:**
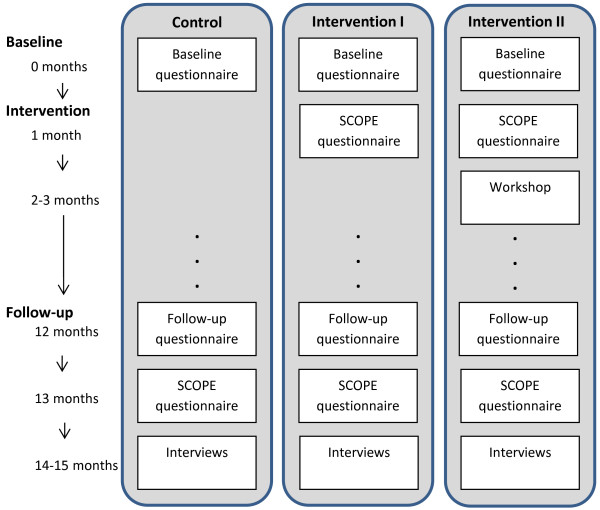
Flowchart of study procedure.

In Figure 2 an overview is given of the intervention procedure and timeframe. Practices in the control arm continue work as usual. All practices are asked to complete a baseline and follow-up questionnaire. At follow-up we administer the SCOPE questionnaire to all participating practices and we will carry out interviews.

#### Intervention arm I

Practices allocated to intervention I receive access to the online SCOPE questionnaire and simultaneously receive a key to download their results in a feedback report. Also, it is communicated that they are expected to anticipate on these results themselves. After one week a reminder is sent. Additionally, one month after the reminder an email is sent reminding the practice of the feedback report and to inform them about the continuation of the study. We interfere as little as possible in this research arm trying to mimic the normal course of events, when a practice would choose for itself to use the SCOPE questionnaire as improvement strategy.

#### Intervention arm II

The practices in intervention II also receive access to the questionnaire. However, these practices do not receive the key to download their results. Instead, they will be given a patient safety workshop at their practice location. The feedback on the results of their questionnaire is embedded in this workshop. The complete feedback report is handed out at the end of the workshop.

### Interventions

The intervention consists of the SCOPE questionnaire solely (intervention I) or the SCOPE combined with a patient safety workshop (intervention II). We chose the SCOPE questionnaire and the workshop for both practical and theoretical reasons. The European Linneaus project recommends the AHRQ safety culture questionnaire, from which the SCOPE has been derived, and the MaPSaF for primary care [17,24]. The SCOPE questionnaire and the Dutch translation of the MaPSaF were both readily available and translated in Dutch. In addition, the tools combine well together as the dimensions largely correspond with each other, facilitating the alignment of the workshop as complementary to the questionnaire.

### SCOPE questionnaire

The SCOPE questionnaire is a culture questionnaire for general practices. The SCOPE is derived from the HSOPS and validated in Dutch general practice [10,25,26]. Chronbach’s alpha ranged between 0.64 - 0.85. The questionnaire consists of 43 items divided over eight dimensions:

(1) Handover and teamwork (8 items)

(2) Support and fellowship (5 items)

(3) Communication openness (6 items)

(4) Feedback about and learning from error (6 items)

(5) Intention to report events (3 items)

(6) Adequate procedures and adequate staffing (7 items)

(7) Overall perceptions of patient safety management (4 items)

(8) Expectations and actions of managers (4 items).

Items are answered using a five point scale varying from ‘strongly disagree’ to ‘strongly agree’ or ‘never’ to ‘always’. In addition, respondents are asked to grade the patient safety culture in their practice. Also, questions on demographics such as gender, age and years of working experience are included. All staff of each practice are asked to complete this questionnaire. For data collection and storage an online system will be used, managed by the Dutch GP Practices Accreditation Organisation [23].

#### SCOPE feedback report

Results of the completed questionnaires are presented in a computerized feedback report. In this report it is presented how often the questionnaire is completed and by which disciplines. Per dimension the percentage positive scores (four or five on the five point-scale) are calculated. When this is 75 percent or higher it is perceived as a “strong” dimension. When this percentage is lower than 50 percent it is perceived as a “weak” dimension and, when scored in between it is “neutral”. The eight dimensions, the percentage positive scores and their classification are presented in a table. Subsequently, all complete dimensions with all questions are reported. Per question the average practice score is reported and compared with an overall benchmark mean that is calculated from data of all practices that have completed the SCOPE questionnaire in 2008 (n = 506-587). When a question scores more than fifteen percent lower than this mean it is depicted red and when higher green. By this, practices can inform themselves on their performance being average, below or above, as compared to the benchmark average. The last page of the report gives general suggestions to improve the patient safety in practice. Suggestions are not fully worked out, but address some issues and refer to more reading material and organizations that can be consulted, if desired. The feedback report is downloaded from the same webpage as where the questionnaires are completed.

### Workshop

The workshop is based on the Dutch translation of the MaPSaF [27]. The MaPSaF is a matrix of nine dimensions in which for each dimension all five maturity stages (pathological, reactive, bureaucratic, proactive, generative) of patient safety are described. We add items on theory on patient safety, human factors engineering and safety culture.

The workshops are organized at each practice location to make it easier for staff to attend. It requires three and a half hour and at least 75% of the staff should be present.

The workshops are given by both an educational scientist who also is a GP and one of the researchers (NV). We intentionally chose for one of the trainers to be an outsider of the research project as well as to be a GP. The first feature allows for questioning and interpreting independently of the research project. The second feature, the trainer being a GP, may allow more rapidly gaining a certain level of understanding and trust among the participants because of being familiar with the GP’s practice and context. We believe that the content of the workshop will be better conveyed when explained by a GP. In addition, as the dialogues can contain intimate content, for example when discussing an incident or flaws in communication between staff, a GP as trainer may be easier to confide in and can also display more understanding of the situation. The researcher attending the workshop gives the opportunity to observe and gather research data. Being part of the research setting is linked with intimate knowledge of the situation, which is essential to develop an understanding ‘from within’.

#### Workshop programme

 ▪ Introduction to patient safety

 – Discussing patient safety terminology

 – Data on number of incidents internationally and nationally

▪ Human factor engineering

 – Why do people make mistakes

 – Interactive examples

 – System approach

▪ Classify organization according to the MaPSaF vignettes on two dimensions (individually)

 – Each respondent classified the maturity of their practice for two dimensions without consultation

▪ Patient safety culture

 – Theory on patient safety culture

▪ Feedback on SCOPE questionnaire

 – Discussion about results

▪ Dialogue about own patient safety culture based on vignettes

 – Vignettes are discussed in pairs (trying to align with each other)

 – Vignettes are discussed with all staff

▪ Brainstorm on possible improvement actions

▪ Drafting of practice improvement action plan

▪ Evaluation & take home message

The workshop is both theoretical and interactive, facilitating discussion among practice staff about their own safety culture. In consecutive order we cover an introduction to patient safety, including discussion about terminology and international and national data about patient safety incident numbers. Followed by theory and interactive examples of human factor engineering and a systems approach to error. Subsequently, we ask all staff to classify the maturity stage, following the MaPSaF matrix, on two vignettes. A vignette is an A4-paper with one dimension worked out in five descriptions of this patient safety theme according to the five stadia. Staff are asked to choose the description that resembles their own daily practice the most. Per workshop we discuss two dimensions: the dimensions that scores the lowest on the SCOPE questionnaire. To be sure the same vignettes will be used for the same SCOPE dimension we made a compatibility table of the SCOPE dimensions and the MaPSaF dimensions (Table 1). The first two SCOPE dimensions have the same MaPSaF dimension. When these are the weakest dimensions, we will use the three weakest SCOPE dimensions in order to have two different vignettes to discuss. The two vignettes in each practice will be different dependent on their SCOPE results.

**Table 1:** Compatibility table of SCOPE questionnaire and MaPSaF dimensions

**SCOPE**	MaPSaF*
Handover and teamwork	Teamwork
Support and fellowship	Teamwork
Communication openness	Communication about patient safety
Feedback and learning from error	Learning from errors and achievement of improvement
Intention to report events	Registration and evaluation of errors
Adequate procedures and adequate staffing	Personnel management and safety issues (Resources)
Overall perceptions of patients safety management	Priority given to patient safety (Staff education and training aimed at patient safety)
Expectations and actions of managers	Errors and responsibility for patient safety

*Dutch version of MaPSaF.

After scoring these vignettes we introduce theory on culture as an important aspect of patient safety. Subsequently, we show the practice results on their SCOPE questionnaires at dimension levels and ask whether these are recognized and discuss these. Next, we will ask to discuss the MaPSaF vignettes in pairs and subsequently in the whole group. During the workshop we facilitate discussion about the patient safety culture of the practice using the results of the SCOPE, the vignettes and other themes that emerge, leading to a brainstorm of possible improvements. At the end of the workshop the staff will draw a practice specific improvement plan. The workshop ends with an evaluation and a round with take home messages from everyone.

#### Pretesting workshop

The workshop has been piloted during a training day in six general practices. The aim of this pilot was twofold, first to evaluate the workshop and to be able to customize possible improvements. Second, to give the trainers a chance to get acquainted to the programme. The workshop was well received, main adjustments were to print out a format for the action plan to take home and to print out the feedback report and handing them out directly after the workshop instead of e-mailing them afterwards.

#### Facultative workshops after ending the study

During the recruiting of the practices, we will communicate that a facultative workshop will be offered for all practices allocated to the control and intervention I arm after ending the study. Hereby, we aim to prevent selective drop-out of practices in the control and intervention I arm.

### Measurements

#### a. Patient safety behaviour

The primary endpoint is the number of incident reports reported by staff in the practice. This endpoint is chosen as incident reporting gives an indication of the patient safety behaviour and culture in the practice. We hypothesize that an increase of reported incidents corresponds to an open patient safety culture. Secondary endpoints are the presence of quality and safety indicators in the previous year such as ‘the presence of a procedure for complaints’, ‘how often safety was on the agenda and/or discussed during team meetings’, ‘whether a safety management policy was present’ and ‘whether safety was subject of staff education’. These outcomes are measured using a practice questionnaire at baseline and follow-up in all study arms.

#### b. Patient safety culture

As stated in the introduction, a culture questionnaire may have an effect on patient safety culture. Therefore, we deploy the SCOPE questionnaire as an intervention. However, as the SCOPE questionnaire will also provide information about the prevailing culture simultaneously, we do not refrain from interpreting and using this data for analyses. Practices in the intervention arms will complete the questionnaire at the beginning of the study as a (part of the) intervention and at follow-up as a measurement tool. The practices in the control arm will only complete the SCOPE questionnaire at follow-up as measurement tool. As such, data on the development of culture will be available for the intervention groups and differences between groups will be available at follow-up.

#### c. Process evaluation

Besides the effect of the intervention we want to examine the implementation process. As a complex intervention is dependent on contextual factors we want to study these in depth to be able to address facilitators and barriers of the intervention. Therefore, we conduct interviews with the physicians and other staff of the practice.

Interviews are conducted by a semi-structured format using a topic list. Topics will examine the patient safety behaviour and culture. First the actual activities are assessed in reference to the research arm where the practice is allocated. Subsequently, patient safety themes that come up during the interview are scrutinized. For example, practices in intervention II will be questioned on their follow-up of the action plan that has been drawn during the workshop. Which activities are implemented and to which level? How did they approach this activity, and what were barriers and facilitators? Practices in the control arm will be questioned on how they perceive patient safety and on what they actually do in their practice around this theme. Interviews are held in an iterative design, by two interviewers. Every week the interviewers will discuss their data briefly to evaluate and, if necessary, to adjust the topic list accordingly [28].

### Statistical power

The power calculation for the effect of the interventions on patient safety behaviour is based on the primary outcome, incident reporting, and resulted in a power of 0.90. The following assumptions are used: 30 practices divided in three equal groups; an improvement of reported incidents in a year (from 50 [13] to 70 (intervention I) to 100 (intervention II) incidents per practice, standard deviation of 30 and an alpha of 0.05.

### Ethical approval

The Medical Research Ethics Committee of the University Medical Center Utrecht concluded that the Medical Research Involving Human Subjects Acts does not apply.

### Implementation of study results

The results of the SCOPE Intervention Study will result in a self-employable product with a guideline that can be used by professionals in primary care to improve their patient safety and culture. To transfer the knowledge acquired during this study among general practitioners we will organize a meeting with representatives of the primary care professional associations other than of general practice. Here, we will present our results and discus possible adjustments of the tool and possible additional information needed to shape the workshop so that these professions in primary care, such as physiotherapy and midwifery, can use this tool as well.

### Data analysis

To analyse the number of reported incidents we will use a poisson regression, if necessary, the analysis will be adjusted for over- or underdispersion [29]. Baseline characteristics such as number of incidents, size of the practice and accreditation will be included as confounders. Where possible we will adjust for baseline measurement of patient safety culture. We will describe patient safety behaviour measured by complaints, meetings and other quality and safety indicators and compare baseline with follow-up. The development of patient safety culture in the two intervention groups and differences in culture between the three arms at follow-up will be analysed by mean scores of the dimension using mixed linear models. All analyses will be corrected for clustering within practices. If necessary a multiple imputation technique will be used for missing data. Data collected from staff during the interviews will be transcribed and analysed with thematic content analysis using software NVivo to code and analyse the data [30].

## Discussion

The purpose of this paper is to outline the rationale and design of the SCOPE Intervention Study. This study will provide insight in the effect of conducting a safety culture questionnaire with a feedback report, on patient safety behaviour and culture in general practice. In addition, this study will reveal whether a complementary workshop to a patient safety culture questionnaire adds to the effect on safety behaviour in general practice. Lastly, interviews will shed a light on the implementation process of the interventions.

This study has several strengths. The SCOPE Intervention Study is one of the first studies that examines the effect of an intervention in primary care on patient safety behaviour and culture. Moreover, the design, a controlled trial, will provide more trustworthy results than previous studies which were observational. Another strength is that the second part of the design is qualitative and will shed light on the implementation process of the interventions. By conducting interviews with practice staff we will gain a deeper understanding on how the interventions work.

Several limitations have to be considered also. Firstly, we ask practices to voluntary participate in our study. This may lead to selection bias. For instance, it is likely that the most motivated practices will decide to participate. However, in daily practice forerunners will also be the first to implement patient safety improvements. By studying the effects and implementation of such interventions we hope to facilitate broader implementation. Secondly, we realise that the number of incidents as outcome is ambiguous as both increasing and decreasing numbers could indicate an improvement in patient safety culture and behaviour. However, we believe that reporting incidents is a good measurement of the change in patient safety. Especially in an organisation where patient safety initiatives are relatively new and the number of incident reports are likely to raise before they will lessen [31]. As reporting is still very uncommon in general practice, we will consider an increase of reported incidents as an indicator of an ameliorating safety culture. Increased rates will indicate the starting of safe reporting and raised awareness. Lastly, interventions in this study are complex and may have a diffuse effect. This may be difficult to measure quantitatively. Therefore, we designed a study with mixed methods to understand the potential effect. The strength is that results will reflect daily practice and approximates the effect to be attained when this intervention would be employed on a large scale.

This study will contribute to the body of knowledge concerning the effect of patient safety interventions in general practice. This knowledge will enhance implementation of patient safety tools in general practice and other primary care professions.

## Competing interests

The authors declare that they have no competing interests.

## Authors’ contributions

DZ, ML and NV designed the study and CW and TV contributed to this design. NV, DZ and ML wrote the manuscript. TV and CW revised the manuscript for important intellectual content. All authors read and approved to the final manuscript.

## Pre-publication history

The pre-publication history for this paper can be accessed here:

http://www.biomedcentral.com/1471-2296/14/127/prepub
